# Retraction: A novel method based on clustering and decision-making for construction project portfolio selection

**DOI:** 10.1371/journal.pone.0354211

**Published:** 2026-07-21

**Authors:** The PLOS One Editors

The *PLOS One* Editors retract this article [1] due to concerns about:

- Compliance with PLOS policy on Artificial Intelligence Tools and Technologies.
- Citation of unretrievable references 1, 2, 4, 5, 8, 10, 12–18, 20–22, 33, 36, 51, 61, 75, and 79.
- The articles cited as References 26 and 72 were retracted before publication of [1].

The first author and the corresponding author acknowledged the concerns and confirmed the use of artificial intelligence tools during manuscript preparation.

MK and AH did not agree with the retraction and stand by the article’s findings. PT either did not respond directly or could not be reached.

## References

[1] Khalilzadeh M, Taebi P, Heidari A. RETRACTED: A novel method based on clustering and decision-making for construction project portfolio selection. PLoS One. 2026;21(3):e0338697. doi: 10.1371/journal.pone.0338697 41774725 PMC12956132

